# Leaf hydraulic decline coordinates stomatal and photosynthetic limitations through anatomical adjustments under drought stress in cotton

**DOI:** 10.3389/fpls.2025.1622308

**Published:** 2025-07-10

**Authors:** Xiuli Li, Shuo Wang, Lingxiao Zhu, Peng Zhang, Hong Qi, Ke Zhang, Hongchun Sun, Yongjiang Zhang, Xiaopeng Lei, Anchang Li, Zhanbiao Wang, Cundong Li, Liantao Liu

**Affiliations:** ^1^ State Key Laboratory of North China Crop Improvement and Regulation, Hebei Agricultural University, Baoding, China; ^2^ Key Laboratory of North China Water-saving Agriculture, Ministry of Agriculture and Rural Affairs, Hebei Agricultural University, Baoding, China; ^3^ Key Laboratory of Crop Growth Regulation of Hebei Province, College of Agronomy, Hebei Agricultural University, Baoding, China; ^4^ Cotton Research Institute, Hebei Academy of Agriculture and Forestry Sciences, Shijiazhuang, China; ^5^ National Key Laboratory of Cotton Bio-breeding and Integrated Utilization, Institute of Cotton Research, Chinese Academy of Agricultural Sciences, Anyang, China

**Keywords:** drought stress, leaf hydraulic conductivity, leaf anatomy, stomatal characteristics, photosynthetic traits

## Abstract

Drought stress detrimentally impacts leaf water transport, lowering transpiration and photosynthetic efficiency and ultimately reducing seed cotton yield. This study investigated the relationship between leaf hydraulic and photosynthetic traits in cotton under three moisture treatments: control (CK), moderate drought (MD), and severe drought (SD). By day 28 after drought stress, drought stress significantly impaired leaf hydraulics, as demonstrated by decreases in leaf hydraulic conductivity (K_leaf_) (9.81% under MD, 12.93% under SD) and leaf water potential (5.79% under MD, 17.54% under SD). Key contributing factors included reduced xylem vessel diameter and number, diminished minor vein density, and decreased aquaporin gene expression. In addition, stomatal width and aperture were significantly reduced with increasing drought severity. Compared with CK, stomatal width and aperture decreased by 6.83% and 13.22% under MD, and by 20.59% and 19.92% under HD. These changes resulted in lower stomatal conductance, net photosynthetic rate, and biomass accumulation, inhibiting growth and reducing plant height, stem diameter, and leaf area. The results of this study provide insights into the anatomical and physiological mechanisms underlying leaf hydraulic conductivity under drought stress.

## 1 Introduction

Drought stress, a major constraint on crop production, impairs plant physiological activity and metabolism, ultimately leading to significant yield losses that threaten agricultural and economic sustainability (Cao et al., 2024). The severity of these impacts depends critically on the intensity, duration, and developmental stage of the stress event (Gray et al., 2016). Cotton is an important economic crop that is prone to drought stress during its growth process, which reduces its yield and quality (Zou et al., 2022). For instance, Bista et al. (2024) found that drought stress led to a reduction in lint and seed cotton yields by 61% and 62%, respectively. Statistically, yield loss caused by drought stress conditions exceeds the sum of losses due to other abiotic stressors (Abdelraheem et al., 2019). Drought stresses commonly result in negative impacts on growth parameters, such as reduced leaf area expansion, declines in the number of nodes and sympodial branches, reduces cotton the number of leaves, and biomass production, and stunted plant height thereby weakening plant growth and potentially leading to irreversible damage (Ahluwalia et al., 2021; Rehman et al., 2022; Zafar et al., 2023). These growth impairments are closely linked to the effects of drought on leaf physiology, which is a critical aspect of the plant’s response to water scarcity.

Drought stress has a profound impact on the physiological and biochemical processes, morphological structure, and overall function of leaves (Seleiman et al., 2021). As the “heart” of the plant, leaves are the primary site for photosynthesis and play a crucial role in the plant’s hydraulic system, serving as a safety valve to mitigate water imbalance (Sack and Scoffoni, 2013). Leaf hydraulic traits function as “regulators” of water transport within the leaf (Li et al., 2015) and are influenced by environmental factors. These traits mediate leaf gas exchange and overall water transport throughout the plant (Villagra et al., 2013). Leaf hydraulic conductivity (K_leaf_), the water flow rate through a leaf at a given time and water potential gradient, reflects the water transport efficiency of the leaf and is a core indicator of leaf hydraulic traits (Sack and Frole, 2006). Studies have shown that K_leaf_ is affected by multiple water transport pathways, such as petioles, leaf vein xylem, vascular sheaths, and mesophyll cells, and its dynamics are not consistent among plant species, developmental periods, and environmental factors (Prado and Maurel, 2013). Under normal circumstances, drought stress decreases K_leaf_, and the degree of its decline is positively correlated with stress severity (Lai et al., 2023), causing an imbalance in leaf hydraulic traits (Blackman et al., 2010). Previous studies have shown that the magnitude of K_leaf_ is directly related to xylem vessel diameter (Jafarikouhini and Sinclair, 2023), the degree of embolism (Dayer et al., 2020), tracheid size (Garcia-Forner et al., 2021), and cell wall thickness (Nardini et al., 2005). These leaf hydraulic traits play a pivotal role in a plant’s ability to adapt to drought conditions. Therefore, studying changes in leaf hydraulic characteristics under drought stress is of significant practical importance. Understanding these changes can reveal how plants adjust their leaf structure and function to cope with drought stress, thereby enhancing their drought resistance and improving growth performance.

Drought stress severely compromises the physiological and anatomical attributes of leaves, diminishing photosynthetic efficiency and water transport capabilities (Zafar et al., 2023). Characterizing these responses is vital for devising strategies to bolster drought resilience and sustain yield and quality in water-scarce environments. K_leaf_ and associated anatomical features, such as xylem vessel dimensions, are pivotal in determining water transport efficiency (Sack and Frole, 2006). Clarifying how drought stress impacts these traits can pinpoint genetic or agronomic interventions that enhance water use efficiency and preserve photosynthetic function. Moreover, stomatal traits, which are intricately connected to leaf hydraulics, regulate water loss and CO_2_ uptake, thereby influencing photosynthesis and plant growth (Ru et al., 2024). Thus, exploring the interplay between leaf hydraulic traits and stomatal characteristics under drought stress can uncover key mechanisms of drought adaptation in cotton. This understanding is crucial for breeding drought-resistant cotton varieties and optimizing irrigation practices to ensure sustainable production in drought-prone areas.

The integrity of the leaf hydraulic system is intrinsically linked to the functionality of leaves, exerting a profound influence on overall plant growth (Ziegler et al., 2023). Drought stress poses a significant challenge to plants by diminishing leaf photosynthetic capacity, which is a primary constraint on crop biomass accumulation and yield (Langhansová et al., 2024; Ru et al., 2024). K_leaf_ is a pivotal factor affecting the photosynthetic capacity of leaves, which are the principal sites of photosynthesis. Li et al. (2021a) found significant positive correlations among the net photosynthetic rate, stomatal conductance, transpiration rate, and leaf hydraulic conductivity in tomato, regardless of whether the plants were under normal water conditions or drought stress. Similarly, in cotton, leaf hydraulic traits are crucial in the plant’s drought response by modulating the efficiency of water transport and stomatal regulation, which are essential for sustaining photosynthesis and biomass production (Lai et al., 2024). When drought stress decreases leaf hydraulic conductivity, it triggers a cascade of physiological responses, including stomatal closure (Ru et al., 2024). This closure reduces stomatal conductance and the net photosynthetic rate, thereby affecting the plant’s ability to convert light energy into chemical energy, which is fundamental for growth and development (Wang et al., 2018; Ye et al., 2021). Building on these insights, our study aims to explore the relationship between leaf hydraulic traits and photosynthetic capacity under drought stress conditions, offering a deeper understanding of the mechanisms that cotton employs to cope with water scarcity.

Although the relationship between K_leaf_ and leaf photosynthetic capacity has been investigated, most related studies have focused on grasses. There is a gap in research on Malvaceae plants, especially cotton. Moreover, the relationship between leaf hydraulic traits and leaf photosynthetic traits remains poorly understood in cotton under drought stress. Therefore, the aims of the present study were (1) to explore the response pattern of leaf hydraulic conductivity to drought stress in cotton; (2) to clarify the anatomical mechanisms by which drought stress regulates leaf hydraulic conductivity; and (3) to determine the relationship between leaf hydraulic conductivity and leaf photosynthetic function under drought stress.

## 2 Materials and methods

### 2.1 Plant material and growth conditions

The experiment was carried out in the intelligent greenhouse of the College of Agronomy, Hebei Agricultural University, in 2023 (Hebei, China). Upland cotton (*Gossypium hirsutum* L.) variety ‘Guoxin Cotton No. 9’ was used as the experimental material, and the seeds were provided by the General Union of Rural Technical Services of Guoxin (Hebei, China). The seeds were soaked in an incubator at 25°C for 24 h. After the seeds showed white tips, they were sown in white PVC culture pots (with a diameter of 10 cm, a height of 20 cm, and a volume of 1.6 L) filled with 2.5 kg of culture medium (with a volume ratio of soil to sand of 3:1), containing 16.93 g kg^−1^ organic matter, 94.60 mg kg^−1^ alkaline dissolved nitrogen, 25.33 mg kg^−1^ effective phosphorus, and 202.07 mg kg^−1^ effective potassium. Three seeds were sown in each culture pot, and only one cotton seedling was retained after one true leaf emerged.

### 2.2 Experimental design

The experimental design employed a randomized complete block design. When the third true leaves of the cotton seedlings were fully expanded, water treatments were initiated: control (CK), moderate drought (MD), and severe drought (SD), with relative water contents of 70–75%, 55–60%, and 40–45%, respectively (Song et al., 2023). Each treatment was replicated in 70 pots. The soil moisture content was monitored by the weighing method every day, and each pot was supplemented with water to reach the set moisture content. The relative humidity in the culture room was constant at (70 ± 5) %. The light intensity was 600 μmol·m^−2^·s^−1^, and the photoperiod was 14/10 h, with day and night temperatures of 28°C/20°C.

### 2.3 Measurement of aboveground morphology

The aboveground morphological traits and biomass were measured at 0, 7, 14, 21, and 28 days after the initiation of the water treatments. Three plants were selected for each treatment to determine the following parameters:

Plant height: Measured from the cotyledon node to the apical growing point.
Stem diameter: Measured 1 cm above the cotyledon node using a vernier caliper.
Leaf area: Calculated using the length×width×0.75 method.
Aboveground dry matter mass: Determined after initial fresh weight recording, followed by kill-drying at 105°C for 30 minutes and drying at 80°C until constant weight.

### 2.4 Leaf hydraulic conductivity

After 0, 7, 14, 21, and 28 days of drought treatment, the third leaf from the top was sampled. There were three replicates for each treatment. The leaf hydraulic conductivity was measured using a plant high-pressure flowmeter (HPFM-Gen, Dynamax, Houston, TX, USA) in transient mode on the leaf, retaining a petiole length of 2 cm, with the applied pressure ranging from 0 to 5 kPa s^−1^ and the pressure and flow rate recorded every 2 seconds (Sack, 2002; Sack et al., 2005). The leaf hydraulic conductivity was calculated as follows:


(1) $$\begin{array}{c} \text{Leaf hydraulic conductivity}\\ =\text{Leaf hydraulic conductance}/\text{Leaf area} \end{array}$$

The leaf area was measured using the leaf length and width method, which was calculated based on the leaf area correction factor method (Mao et al., 2014), as follows:


(2) Leaf area=Leaf length×Leaf width×0.75

### 2.5 Leaf water potential

After 0, 7, 14, 21, and 28 days of drought treatment, the third leaf from the top was measured using a portable pressure chamber (PMS670, PMS Instrument Company, USA), with three replicates for each treatment. The leaves were first equilibrated in sealed black plastic bags for 20 min, then cut off at the base of the petiole and placed into a pressure chamber. Subsequently, pressure was slowly applied. The minimum pressure at which the first drop of water was observed exuding from the petiole under observation with a magnifying glass was regarded as the leaf water potential (Ψ_leaf_) (Müllers et al., 2022).

### 2.6 Anatomical leaf and petiole structure

On day 28 post-treatment, the third leaves from the top were collected from each treatment for leaf and petiole anatomical analyses. For leaves, 0.5 cm × 0.5 cm sections were cut perpendicular to the veins, while petioles were cut into 0.5–1 cm segments, starting 1 cm from the leaf. Three replicates per treatment were prepared using the paraffin section method. Samples were fixed with formaldehyde–alcohol–acetic acid (FAA), treated with xylene and absolute ethanol, and then embedded in paraffin (Zhang et al., 2022). After saffron and solid green staining, the sections were sealed and stored at 4°C. Images were captured using a digital microscope (BX53, Olympus, Monolith, Japan) and analyzed using NIS-Elements software. Leaf parameters included thickness, cross-sectional area, and xylem vessel area, number, and diameter. Petiole analyses focused on the cross-sectional area, diameter, and the area and number of xylem vessels.

### 2.7 Leaf vein density

On day 28 post-treatment, the third leaves from the top were scanned (Epson Perfection V39; Epson, Suwa, Japan) with three replicates per treatment. The major (VLA_major_) and minor (VLA_minor_) vein densities were measured. Primary veins were assessed in intact leaves, and secondary veins were assessed in half of them for VLA_major_. Leaves were soaked in 95% ethanol for 1–2 days, stained with 1% saffron, and observed under a microscope (BX53, Olympus) to determine the vein length and field area (Lu et al., 2019). The vein density (VLA_minor_) was calculated using NIS-Elements software, reflecting the length of veins per unit leaf area.

### 2.8 Stomatal size and density

On day 28 post-treatment, the third leaf was selected from the main stems of cotton under each treatment (three replicates per treatment). The abaxial leaf surfaces were lightly coated with nail polish, allowed to dry for 3 min, and transferred onto clean slides using transparent tape. Observations and image acquisition were conducted using a digital microscope (BX53, Olympus, Monolith, Japan). The stomatal area and number were measured using NIS-Elements software, enabling the calculation of stomatal density (number of stomata per unit area).

### 2.9 Leaf gas exchange parameters

On days 0, 7, 14, 21, and 28 post-treatment, the environmental control mode of a portable photosynthesis system (LI-6400, Li-Cor, Lincoln, NE, USA) was adopted to measure the gas exchange parameters, including net photosynthetic rate (A_n_), transpiration rate (E), stomatal conductance (g_s_), and intercellular CO_2_ concentration (C_i_) in the third leaves from the top of cotton plants (Sáez et al., 2018). The light intensity of the red and blue light sources was 500 μmol·m^−2^·s^−1^, and the CO_2_ concentration was 400 μmol·mol^−1^. There were three replicates for each treatment. The instantaneous water use efficiency of the leaves was calculated as follows (Li et al., 2021b):


(3) $${\text{WUE}}_{\text{i}}=\text{E}/{\text{g}}_{\text{s}}$$

### 2.10 Chlorophyll fluorescence parameters of leaves

After 0, 7, 14, 21, and 28 days of drought treatment, a PAM-2500 portable chlorophyll fluorometer (PAM-2500, WALZ, Effeltrich, Germany) was used to measure the maximum photochemical efficiency of photosystem II (PSII; Fv/Fm) and the actual photochemical quantum yield of PSII (ΦPSII) for each treatment. The measurement sites were the same as those for the photosynthesis measurements, and there were three replicates for each treatment.

### 2.11 Aquaporin content and gene expression level

To determine the aquaporin content, the third leaves from the top of cotton seedlings were sampled on days 0, 7, 14, 21, and 28 post-drought treatment (three replicates per treatment). Samples were labeled, wrapped in aluminum foil, rapidly frozen in liquid nitrogen, and stored at –80°C. The aquaporin content was determined using an enzyme-linked immunosorbent assay (ELISA) kit (YJ191258) from Shanghai Yuanju Technology Center, following the manufacturer’s protocol.

To determine the expression of aquaporin genes, 50 mg of the same leaves was collected on day 28 post-treatment, frozen in liquid nitrogen, and stored at −80°C (three replicates per treatment). RNA was extracted, and its concentration and quality were assessed. RNA extraction and cDNA synthesis procedures are detailed in the appendix. All reagents were obtained from Sigma-Aldrich.

### 2.12 Statistical analysis

Data were recorded and organized using Microsoft Excel 2010. Statistical analyses were performed with IBM SPSS Statistics 25.0, employing one-way ANOVA followed by Duncan’s multiple comparison tests to assess significance. Graphs were plotted using GraphPad Prism 8.0, and correlation analyses were conducted with Origin 2023.

## 3 Results

### 3.1 Growth, photosynthetic, and fluorescence traits of cotton plants under drought stress

**Figure 1:**
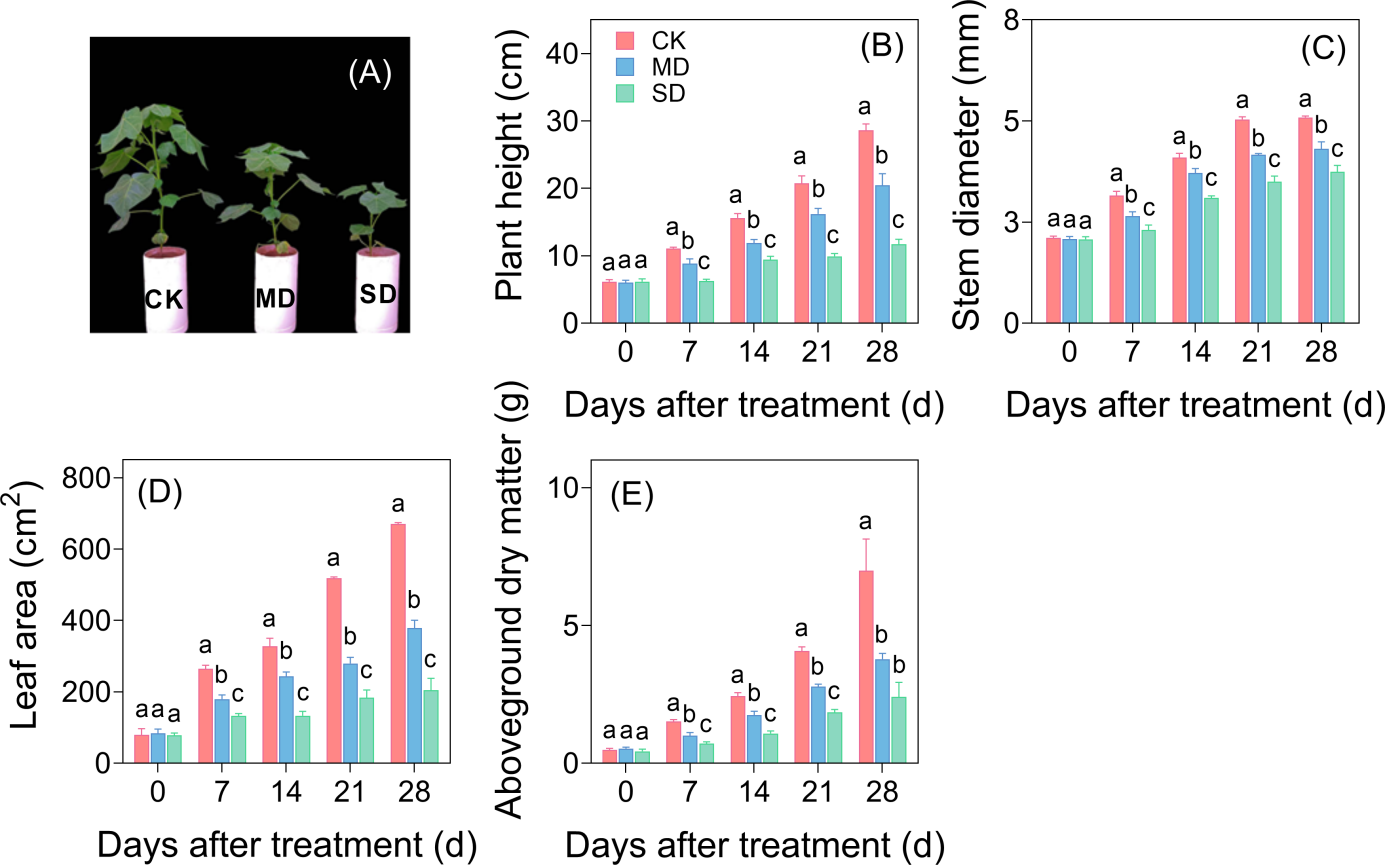
Effects of drought stress on aboveground morphology **(A)**, plant height **(B)**, stem diameter **(C)**, leaf area **(D)**, and aboveground dry matter **(E)** of cotton seedlings. Values are the mean ± SD (n = 3). Different lowercase letters indicate significant differences according to Duncan’s method (*P*< 0.05).

Drought stress exhibited progressive inhibitory effects on cotton seedling growth with increasing drought intensity and duration (**Figure 1A**). Quantitative analysis revealed significant reductions in key growth parameters, including plant height, stem diameter, leaf area, and aboveground dry matter mass. After 28 days of drought treatment, MD decreased these parameters by 28.52%, 15.32%, 43.51%, and 45.92%, respectively, compared with CK. SD induced more pronounced growth suppression, with corresponding reductions reaching 59.14% in plant height, 26.52% in stem diameter, 69.49% in leaf area, and 65.47% in aboveground dry matter mass (**Figures 1B–E**).

**Figure 2:**
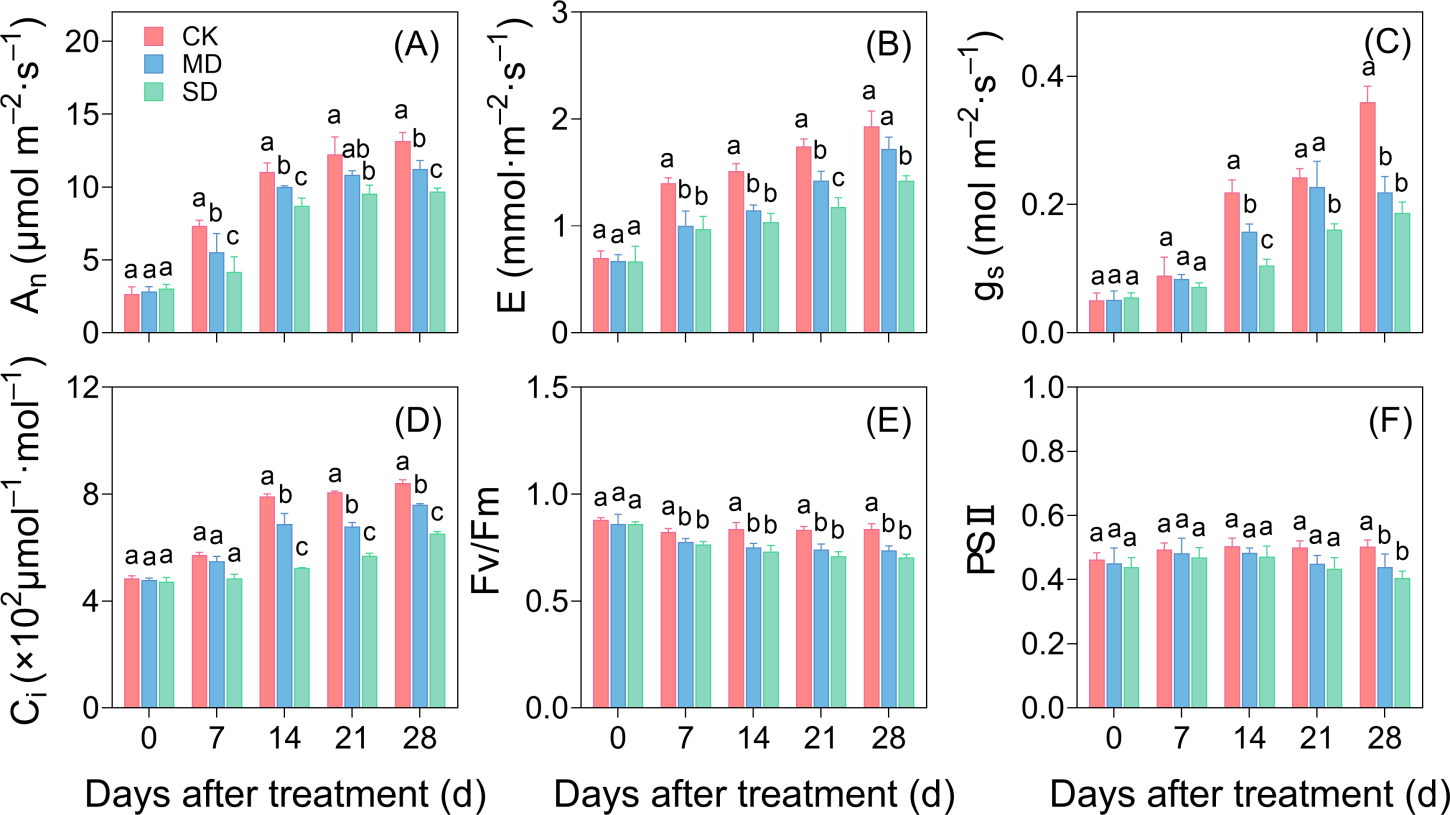
Effects of drought stress on photosynthetic fluorescence of cotton at different times. Effects of drought stress on the net photosynthetic rate **(A)**, transpiration rate **(B)**, stomatal conductance **(C)**, intercellular carbon dioxide concentration **(D)**, and chlorophyllin II fluorescence parameters in functional leaves from the main stem **(E, F)** of cotton seedlings. Values are the mean ± SD (n = 3). Different lowercase letters indicate significant differences according to Duncan’s method (*P*< 0.05).

A_n_ and E of cotton seedlings that underwent drought treatment were substantially lower than those of the CK group starting after 7 days of treatment. This difference augmented progressively over time (**Figures 2A, B**). On day 28 of drought stress, g_s_, A_n_, E, and C_i_ in the MD treatment had diminished by 39.07%, 14.68%, 10.88%, and 9.88%, respectively. In the SD treatment, these values decreased by 48.11%, 26.39%, 26.42%, and 22.69%, respectively (**Figures 2A–D**). The Fv/Fm and ΦPSII values of the leaves under drought stress conditions were lower than those in CK. On day 28 of drought stress, Fv/Fm of the leaves in the MD and SD treatments was significantly reduced by 11.95% and 15.94%, respectively, compared with CK (**Figure 2E**). Similarly, ΦPSII decreased significantly by 12.67% and 20.72% in the MD and SD treatments, respectively (**Figure 2F**).

### 3.2 Effect of drought stress on hydraulic traits of cotton plants

**Figure 3:**
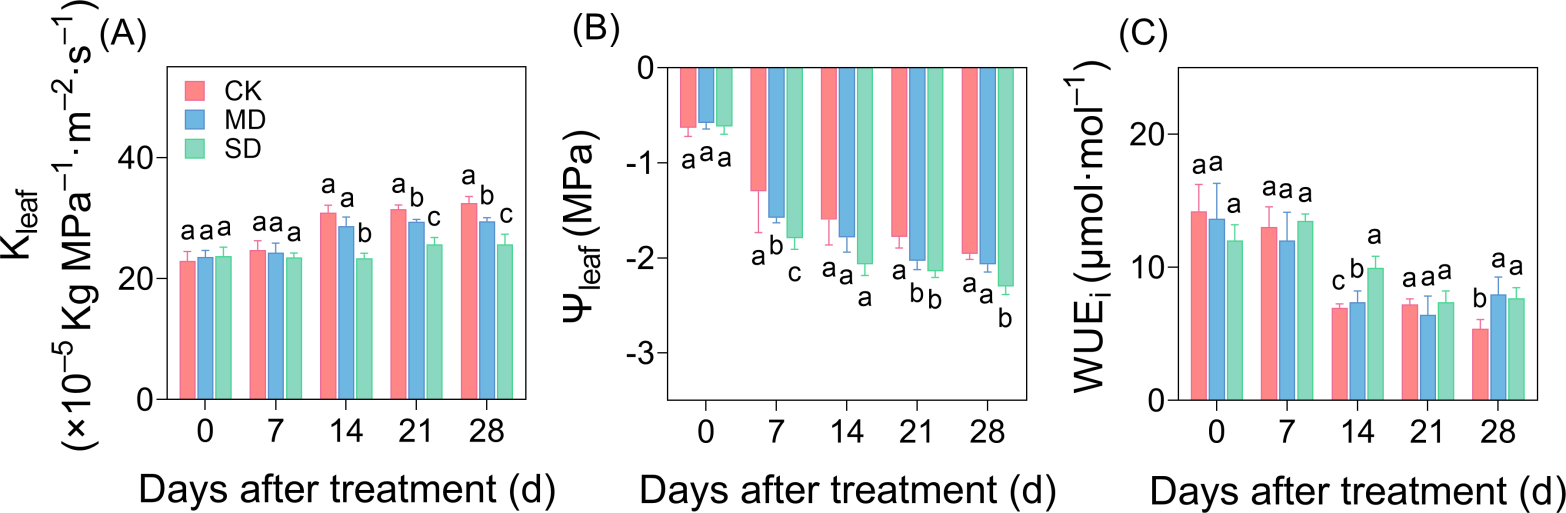
Effect of drought stress on leaf hydraulic traits over time in cotton. Effects of drought stress on hydraulic conductivity **(A)**, water potential **(B)**, and instantaneous water use efficiency **(C)** of cotton seedlings. Values are the mean ± SD (n = 3). Different lowercase letters indicate significant differences according to Duncan’s method (*P*< 0.05).

As the degree and duration of drought stress increased, K_leaf_ showed a decreasing trend compared with CK (**Figure 3A**), and by day 14 of drought treatment, a significant difference was observed, with K_leaf_ reduced by 7.17% and 18.77% in the MD and SD treatments, respectively, compared with CK. On days 21 and 28 of drought stress, K_leaf_ decreased by 6.54% and 9.18%, respectively, in the MD treatment and by 12.77% and 12.93%, respectively, in the SD treatment compared with CK (**Figure 3A**). Differences in Ψ_leaf_ were observed among treatments after 7 days of drought stress. Drought stress accelerated the decline in leaf water potential, and on day 28, leaf water potential in the SD treatment decreased significantly by 17.55% compared with CK (**Figure 3B**). On day 28 of drought stress, WUE_i_ increased significantly by 47.42% and 42.28% in the MD and SD treatments, respectively, compared with CK (**Figure 3C**).

### 3.3 Effect of drought stress on aquaporins in cotton plants

**Figure 4:**
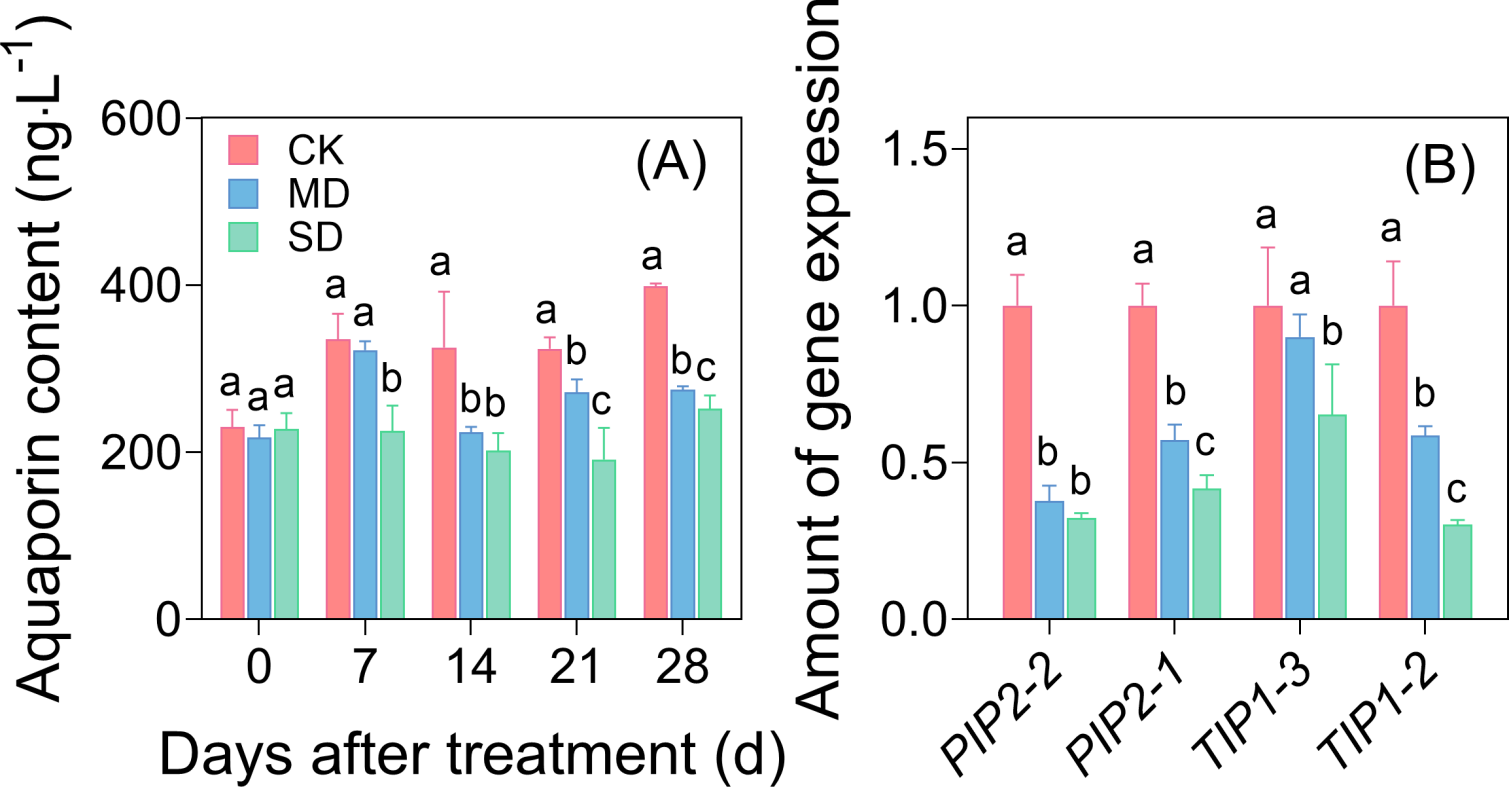
Effects of drought stress on aquaporin content **(A)** and the expression of aquaporin synthesis genes **(B)** in cotton seedling leaves. Values are the mean ± SD (n = 3). Different lowercase letters indicate significant differences according to Duncan’s method (*P*< 0.05).

After 7 days of drought stress, significant differences in aquaporin content were observed among the treatments (**Figure 4A**). By day 28 of drought stress, the aquaporin content in the MD and SD treatments had decreased by 31.07% and 36.73%, respectively, compared with the CK treatment (**Figure 4A**). Further analysis of the synthesized genes of water channel proteins showed that *GhPIP2-1*, *GhPIP2-2*, *GhTIP1-2*, and *GhTIP1-3* were significantly downregulated under drought stress. The expression of *GhPIP2-1*, *GhPIP2-2*, and *GhTIP1-2* was downregulated 2–2.5-fold (**Figure 4B**).

### 3.4 Effect of drought stress on anatomical traits in cotton

#### 3.4.1 Anatomical traits of leaves and petioles of cotton plants under drought stress

**Figure 5:**
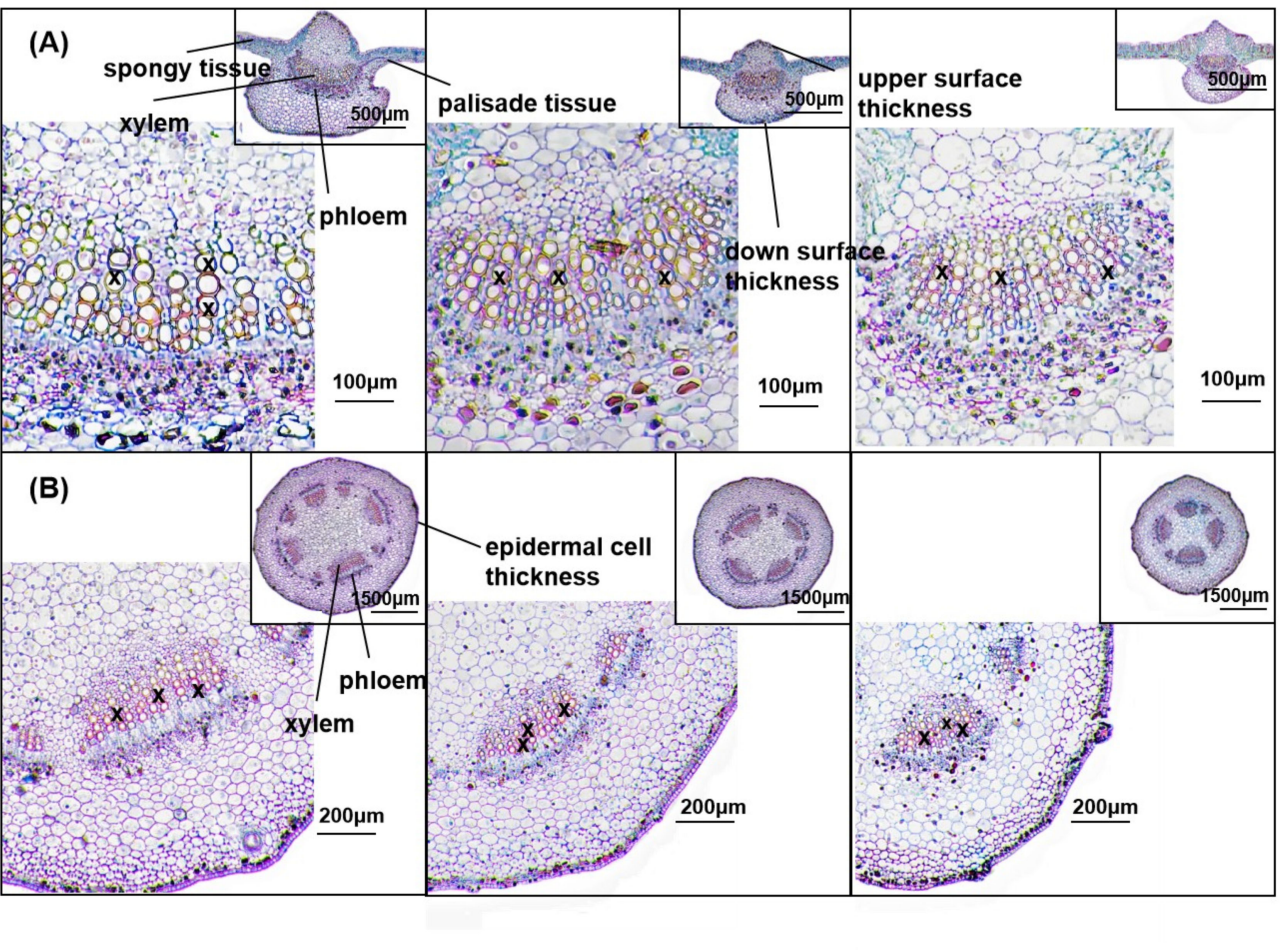
Effect of drought stress on plant anatomical traits. Paraffin sections of cotton leaves in CK, MD, and SD **(A)** and petioles in CK, MD, and SD **(B)** were evaluated after 28 days of drought stress. In **(A)**, the image shows the petiole anatomy observed at 40× magnification, and the inset image in the upper right corner shows the complete leaf anatomy observed at 20× magnification. In **(B)**, the image shows the petiole anatomy observed at 40× magnification, and the inset image in the upper right corner shows the complete petiole anatomy observed at 10× magnification. X marks the xylem vessels.

**Figure 5** shows the anatomical structures of petioles (A) and leaves (B) on day 28 of drought treatment. Compared with CK, the cross-sectional area, phloem area, xylem area, and epidermal cell thickness of petioles were significantly reduced in the MD and SD treatments. Under MD treatment, these parameters decreased by 27.42%, 40.61%, 41.08%, and 10.68%, respectively, while under SD treatment, they decreased by 15.55%, 44.46%, 56.26%, and 12.37%, respectively. The diameter of the xylem vessels under MD treatment was significantly reduced by 17.61% compared with CK. Under SD treatment, the number and diameter of xylem vessels were significantly reduced by 10.45% and 18.83%, respectively, compared with CK (**Table 1**).

**Table 1:** Effect of drought stress on anatomical structure of petiole of cotton seedlings.

Treatments	CK	MD	SD
Cross Sectional Area (10^6^ µm^2^)	7.04 ± 0.13a	5.11 ± 0.36b	3.56 ± 0.03c
Area of phloem(10^5^ µm^2^)	6.50 ± 0.98a	3.86 ± 0.27b	3.61 ± 0.15b
Area of Xylem(10^5^ µm^2^)	12.39 ± 1.59a	7.30 ± 0.51b	5.42 ± 0.38b
Epidermal cell thickness(µm)	18.91 ± 0.71a	16.57 ± 0.21b	16.89 ± 0.45b
Number of xylem vessels	217.00 ± 2.65a	207.33 ± 6.03a	194.33 ± 8.96b
Xylem vessel diameter(µm)	27.36 ± 1.49a	23.26 ± 1.04b	22.21 ± 0.76b

Values are the mean ± SD (n=3). Different lowercase letters indicate significant differences according to Duncan’s method (*P*<0.05).

**Table 2** shows that significant changes occurred in leaf anatomical parameters under drought treatments. Under MD treatment, the upper epidermal cell thickness, spongy tissue thickness, xylem area, phloem area, number of xylem vessels, and xylem vessel diameter decreased by 12.41%, 6.60%, 61.51%, 65.84%, 20.27%, and 18.84%, respectively. Under SD treatment, these parameters decreased more substantially, with reductions of 36.29%, 17.99%, 68.34%, 72.48%, 27.07%, and 38.21%, respectively. Compared with CK, the thickness of the lower epidermal cells and palisade tissue significantly increased by 29.09% and 13.08%, respectively, under MD treatment, and by 43.60% and 9.58%, respectively, under SD treatment.

**Table 2:** Effects of drought stress on anatomical structure of cotton seedling leaves.

Treatments	CK	MD	SD
Thickness of upper epidermis (µm)	23.70 ± 0.67a	20.76 ± 0.61b	15.10 ± 0.97c
Thickness of lower epidermis (µm)	10.38 ± 0.50c	13.40 ± 0.72b	14.85 ± 0.82a
Thickness of Palisade tissue (µm)	68.97 ± 0.65b	77.99 ± 1.81a	75.58 ± 2.44a
Thickness of Spongy tissue (µm)	108.51 ± 3.45a	101.35 ± 2.84b	88.99 ± 2.84c
Area of Xylem(10^4^ µm^2^)	25.33 ± 0.88a	9.75 ± 0.44b	8.02 ± 0.16c
Area of phloem(10^4^ µm^2^)	20.20 ± 1.56a	6.90 ± 0.17b	5.56 ± 0.14b
Number of xylem vessels	120.67 ± 6.66a	100.33 ± 3.21b	88.00 ± 4.58c
Xylem vessel diameter(µm)	25.38 ± 1.42a	21.36 ± 1.66b	15.68 ± 0.81c

Values are the mean ± SD (n=3). Different lowercase letters indicate significant differences according to Duncan’s method (*P*<0.05).

#### 3.4.2 Effect of drought stress on stomatal characteristics of cotton plants

**Figure 6:**
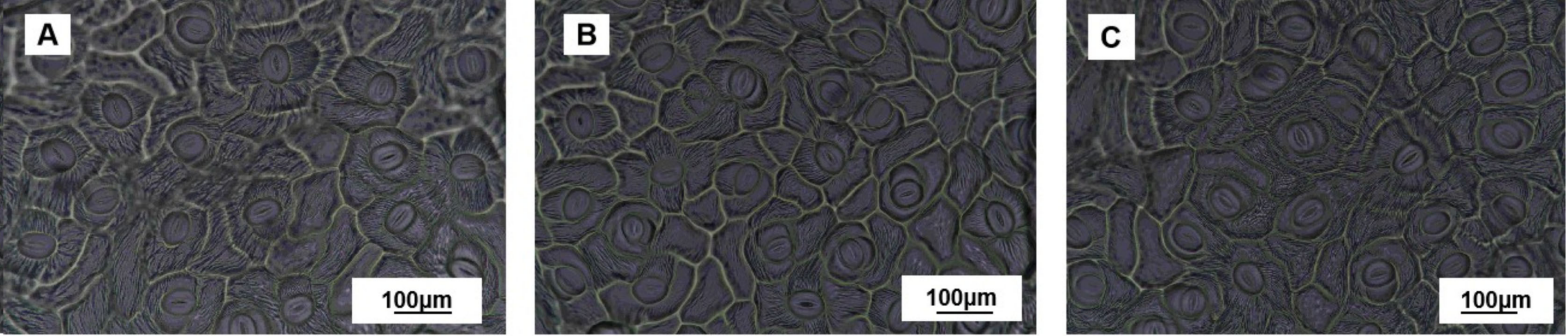
Measurement of stomata in the control **(A)**, mild drought treatment **(B)**, and severe drought treatment groups **(C)** after 28 days of drought treatment. Values are the mean ± SD (n = 3). Different lowercase letters indicate significant differences according to Duncan’s method (*P*< 0.05).

**Figure 6** shows the stomatal images after 28 days of drought treatment. As shown in **Table 3**, stomatal width, stomatal area, and stomatal aperture decreased by 6.83%, 6.81%, and 13.22%, respectively, under MD treatment compared with CK. This was further aggravated in the SD treatment, with significant decreases of 13.37%, 13.64%, and 19.92%, respectively. Compared with the CK, the values in the MD and SD treatments increased by 18.96% and 24.14%, respectively.

**Table 3:** Effect of drought stress on stomata in cotton seedling leaves.

Treatments	Stomatal length (µm)	Stomatal width (µm)	Stomatal area (µm^2^)	Stomatal density (cm^-2^)	Stomatal aperture (µm)
CK	24.49 ± 0.43a	17.72 ± 0.84a	401.01 ± 20.55a	2.26 ± 0.18b	5.87 ± 0.25a
MD	24.11 ± 1.58a	16.51 ± 0.22b	373.70 ± 27.54ab	2.69 ± 0.12a	5.10 ± 0.10b
SD	22.29 ± 0.64a	15.35 ± 0.16c	346.33 ± 6.67b	2.81 ± 0.23a	4.70 ± 0.19b

Values are the mean ± SD (n=3). Different lowercase letters indicate significant differences according to Duncan’s method (*P*<0.05).

#### 3.4.3 Effect of drought stress on leaf vein characteristics of cotton plants

**Figure 7:**
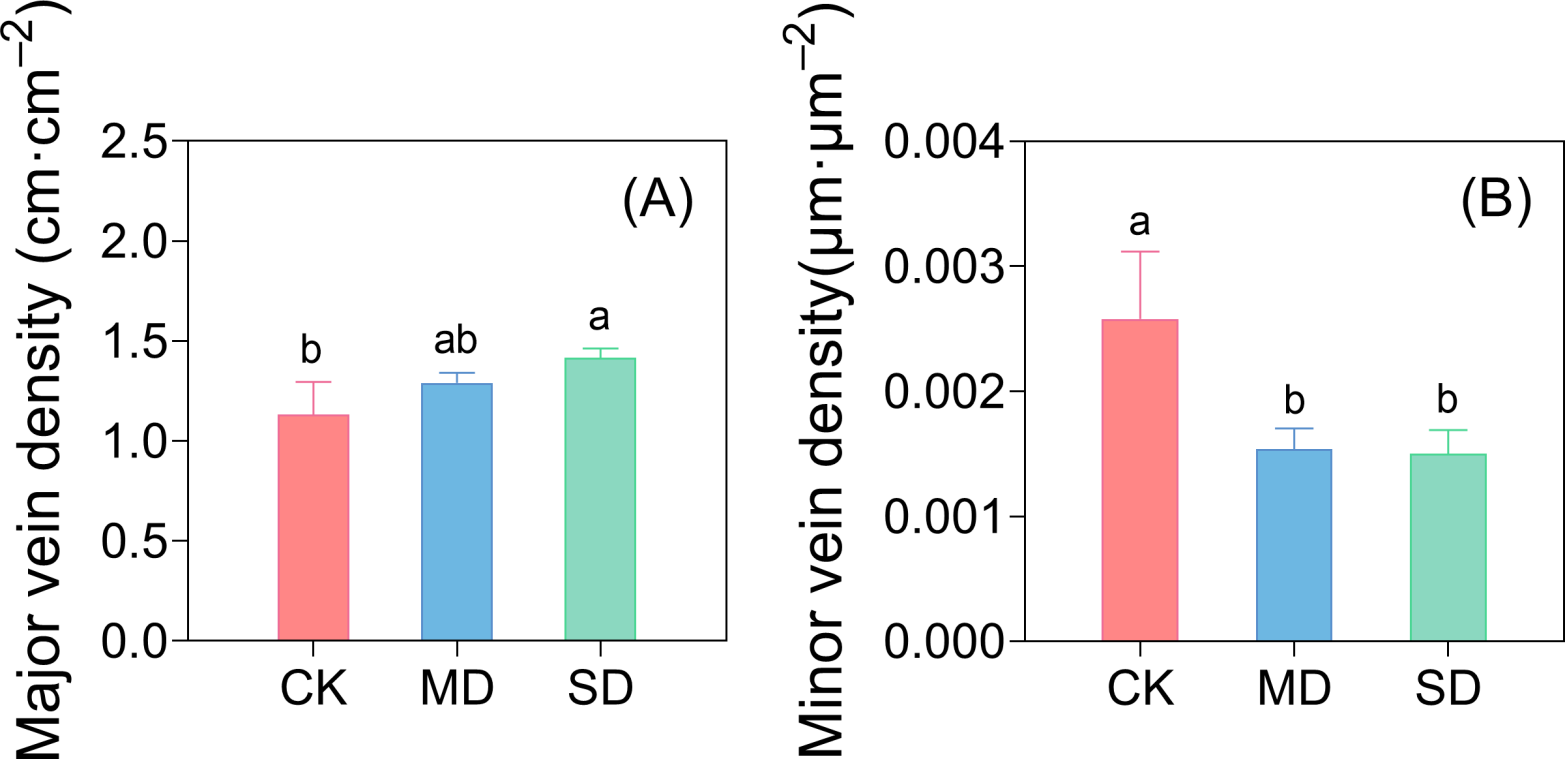
Effect of drought stress on major vein density **(A)** and minor vein density **(B)** in cotton seedlings. Values are means ± SD (n = 3). Different small letters mean significant differences according to the Duncan’s method (*P*< 0.05).

Under SD treatment, VLA_major_ was significantly higher than that under CK (**Figure 7**). Compared with CK, VLA_major_ increased by 14.16% and 25.66% under the MD and SD treatments, respectively. The VLA_minor_ density was significantly lower in the MD and SD treatments than in CK, with decreases of 40.31% and 41.86%, respectively.

### 3.5 Xylem vessel diameter, number, and relationship between VLA_minor_ and K_leaf_

**Figure 8:**
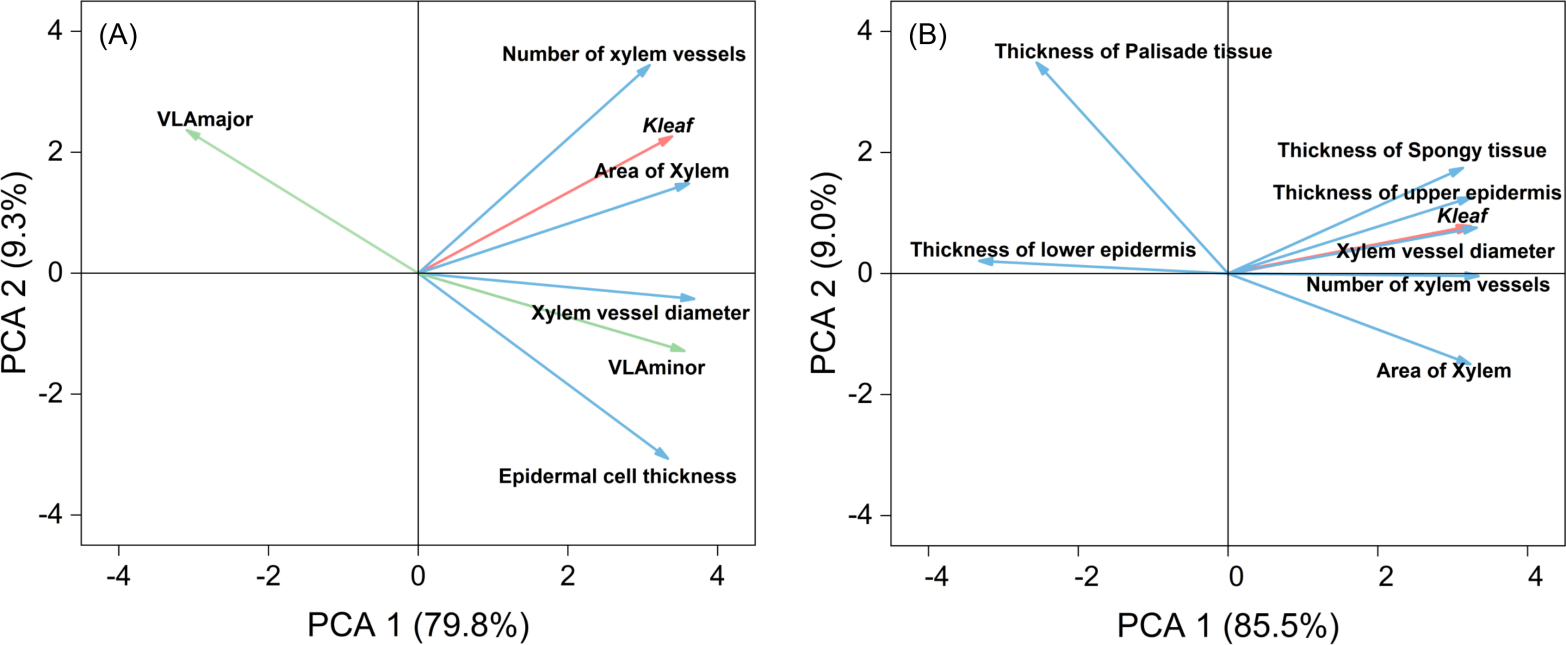
Principal component analysis (PCA) of hydraulic conductivity (red line), anatomical characteristics of the petiole (blue line), and vein density (green line) of leaves using original data **(A)**, and PCA of hydraulic conductivity (red line) and anatomical characteristics of the leaf (blue line) using original data **(B)**. Values in brackets are percentages explained by the first two components.

To identify the primary anatomical factors influencing K_leaf_ under drought stress, we conducted principal component analysis (PCA) on hydraulic conductivity and petiole anatomical traits (**Figure 8A**). The key factors influencing leaf hydraulic conductivity, including main vein density, secondary vein density, number of xylem vessels, xylem vessel diameter, xylem area, and epidermal cell thickness, accounted for 89.10% of the total variance. These factors were negatively correlated with primary vein density, and had extremely significant positive correlations with the number of xylem vessels, xylem vessel diameter, xylem area, and secondary vein density (**Supplementary Table S4**). The main factors affecting K_leaf_ were xylem vessel diameter and xylem area of the petiole.

PCA was conducted on K_leaf_ and leaf anatomical traits (**Figure 8B**). Leaf hydraulic conductivity and leaf anatomical factors accounted for 94.50% of the total variance. K_leaf_ was mainly influenced by the number and diameter of xylem vessels in the leaf, upper epidermal cell thickness in the leaf, spongy tissue thickness, and the number of xylem vessels in the petiole. Moreover, these indicators had extremely significant positive correlations with K_leaf_ (**Supplementary Table S4**). In conclusion, K_leaf_ was mainly affected by the diameter and number of xylem vessels in the leaf and petiole as well as secondary leaf vein density.

### 3.6 K_leaf_ in relation to the photosynthetic function and water status

**Figure 9:**
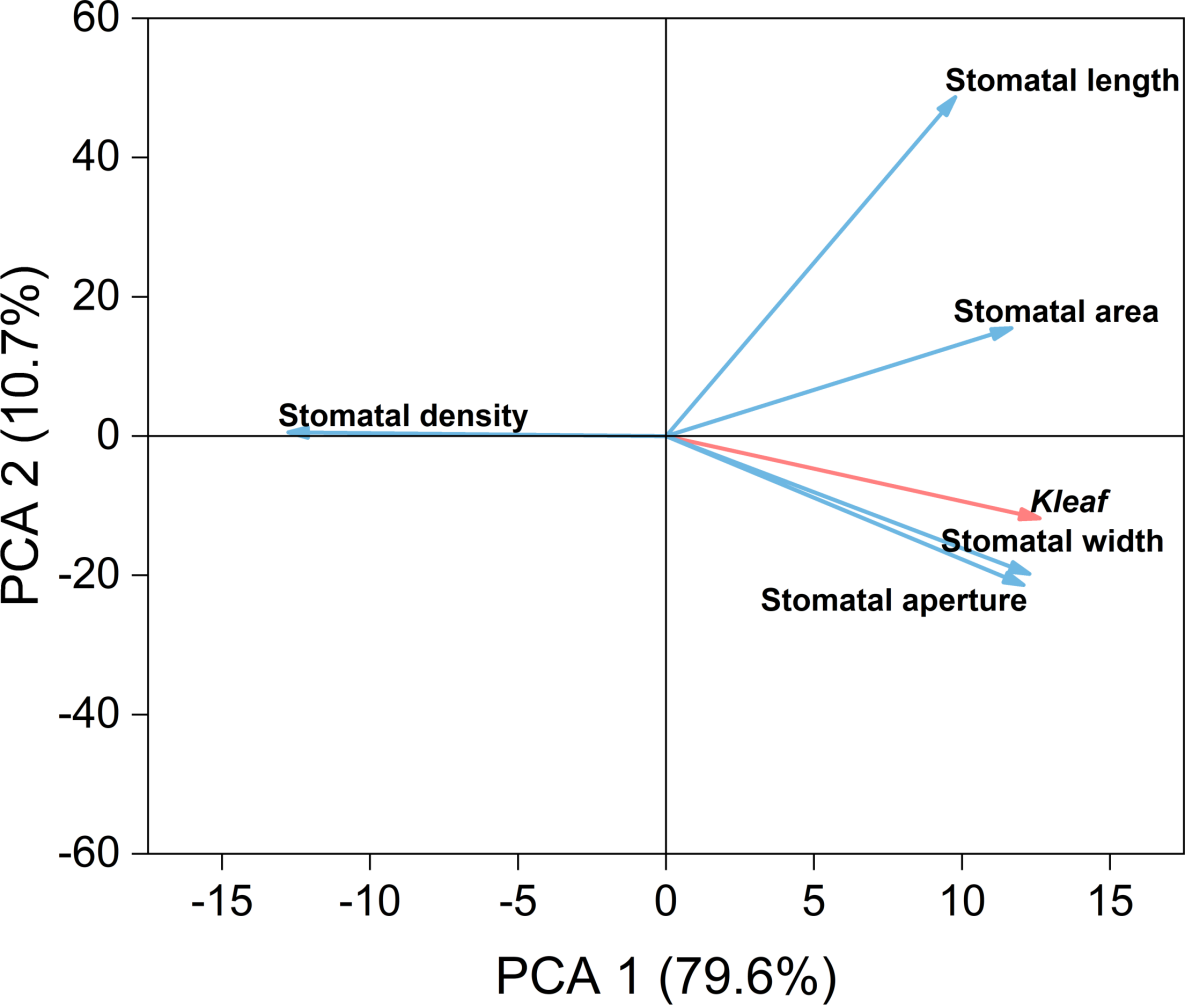
Principal component analysis (PCA) of hydraulic conductivity (red line) and stomatal characteristics (blue line) of leaves using original data. Values in brackets are percentages explained by the first two components.

To better understand the relationships between leaf hydraulic conductivity and stomatal traits, we conducted PCA on the relevant indicators under three water treatments (**Figure 9**). K_leaf_ was significantly negatively correlated with stomatal density and had extremely significant positive correlations with stomatal area, stomatal width, and stomatal aperture. K_leaf_ mainly influenced stomatal width and aperture.

**Figure 10:**
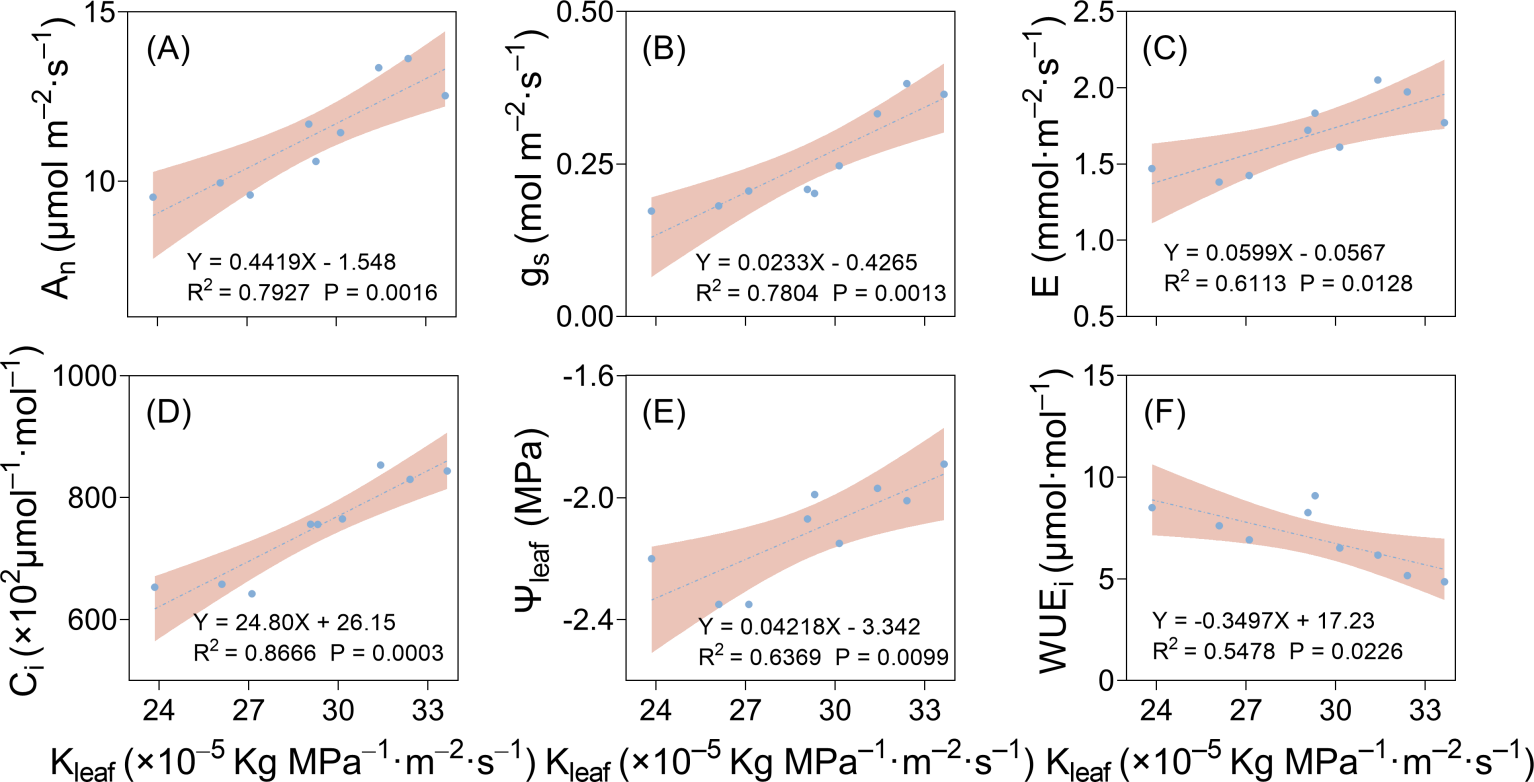
Linear regression results of cotton leaf hydraulic and stomatal conductance **(A)**, net photosynthetic rate **(B)**, transpiration rate **(C)**, intercellular carbon dioxide concentration **(D)**, leaf water potential **(E)**, and instantaneous water use efficiency **(F)** of cotton plants.

Correlation analysis was carried out between leaf hydraulic conductivity and photosynthetic parameters. As shown in **Figure 10**, there were significant positive correlations between K_leaf_ and g_s_, A_n_, E, and C_i_. With the increase in drought stress severity, K_leaf_ decreased, and g_s_, A_n_, E, and C_i_ decreased with the decline in K_leaf_ (**Figures 10A–D**). In conclusion, the decrease in K_leaf_ under drought stress led to the decline in stomatal aperture and width, decreasing g_s_, A_n_, E, and C_i_, attenuating photosynthetic function.

There was a positive correlation between K_leaf_ and leaf water potential (**Figure 10E**) and a negative correlation between K_leaf_ and instantaneous leaf water use efficiency (**Figure 10F**). Therefore, drought stress decreased leaf xylem vessel diameter, number, and K_leaf_, leading to a decrease in leaf water potential and an increase in instantaneous leaf water use efficiency.

## 4 Discussion

### 4.1 Effects of drought stress on hydraulic traits in cotton

Leaf hydraulic dysfunction is a pivotal adaptive response when plants face drought stress, critically influencing plant fitness under water stress conditions (Blackman et al., 2010). Hydraulic traits act as multifaceted functional indices, governing water transport efficiency and stomatal regulation (Creek et al., 2018), and shaping plant ecological strategies in growth dynamics and resource competition (Cosme et al., 2017; Poorter et al., 2017). Previous studies have revealed species-specific drought adaptation mechanisms: rice enhances water-use efficiency by reducing leaf water potential (Yang et al., 2024), while maize prioritizes drought tolerance by decreasing leaf hydraulic conductivity (K_leaf_) (Qiao et al., 2020). These findings align with current experimental evidence suggesting that progressive drought intensification reduces vascular water supply capacity, with synchronously decreasing K_leaf_ (**Figure 3A**) and leaf water potential (**Figure 3B**). This indicates that cotton plants adapt to drought conditions by reducing K_leaf_ and leaf water potential, thereby maintaining normal growth.

### 4.2 Factors affecting K_leaf_ under drought stress

K_leaf_ is a critical hydraulic signal modulated by multifaceted anatomical and molecular factors (Sack and Scoffoni, 2013). Water transport in leaves operates through two sequential pathways: xylem vessels in petioles and vascular bundles, and the post-xylem pathway involving aquaporin-mediated membrane transport. These pathways contribute substantially to total leaf hydraulic resistance, with their coordinated regulation directly determining K_leaf_ (Prado and Maurel, 2013; Kaack et al., 2021). Our findings revealed that drought stress disrupted these pathways synergistically—reducing vein density, xylem vessel diameters and numbers, and aquaporin expression—collectively impairing hydraulic efficiency. This dual pathway suppression provides mechanistic insights into drought-induced K_leaf_ decline.

VLA_minor_ plays a key role in leaf pulp hydraulic transport, especially under drought stress, and its regulatory role significantly affects leaf hydraulic efficiency and photosynthetic capacity. Unlike the VLA_major_, which mainly provides mechanical support and water redundancy, VLA_minor_ is directly involved in water transport between leaf pulp cells, thereby affecting K_leaf_ and gas exchange efficiency (Baird et al., 2021). In this study, drought stress significantly reduced VLA_minor_ (40.31% and 41.86% decrease in MD and SD treatments, respectively), which was significantly and positively correlated with the decrease in K_leaf_ (**Figure 7B**; **Supplementary Figure S1B**). This finding contrasts with the findings in rice, where there was no correlation between VLA_minor_ and K_leaf_ under drought conditions (Xiong et al., 2015; Caringella et al., 2015; Scoffoni et al., 2011), whereas cotton exhibited a significant decrease in VLA_minor_, leading to a decrease in K_leaf_. This difference may stem from the differences in leaf vein structure and water utilization strategies between cotton and rice. Rice, as a monocotyledon, has a parallel leaf vein structure with higher hydraulic redundancy under drought conditions, whereas the reticulate leaf vein structure of cotton is more susceptible to drought-induced embolism and cell wall thickening (Zou et al., 2022). In addition, the decrease in VLA_minor_ may also be related to anatomical changes in cotton chloroplasts, such as a reduction in the thickness of spongy tissue (**Table 2**), which further limits the efficiency of water transport between chloroplasts (Sack and Frole, 2006). Thus, VLA_minor_ is not only a key regulator of cotton leaf hydraulic efficiency, but also an important component of its drought adaptation strategy. Future studies should further explore the potential of increasing VLA_minor_ through genetic improvement or agronomic measures to enhance the hydraulic efficiency and photosynthetic performance of cotton under drought conditions.

Plant water transport relies on the axial xylem vessel system (Cochard et al., 2004). This hydraulic pathway initiates soil water absorption via roots, progressing through root-to-stem xylem networks, petiolar vessels, and leaf vein vessels, ultimately delivering water to mesophyll cells for transpirational loss through stomata. Xylem vessel morphology directly regulates water transport efficiency from stems to foliar tissues (Kaack et al., 2021). Our investigation revealed marked anatomical alterations under drought conditions, including a diminished xylem vessel diameter, frequency, and cross-sectional area in both leaves and petioles (**Figure 5**; **Tables 1**, **2**), which confirmed that stomatal change served as an adaptive strategy for mitigating water loss while maintaining plant viability. These structural modifications highlight the physiological acclimation mechanism in cotton under water deficit.

Previous studies have shown that the morphology of xylem vessels affects K_leaf_. The larger the vessel diameters and the greater their number, the larger the K_leaf_, resulting in enhanced water transport capacity. Conversely, smaller vessel diameters, reduced vessel areas, and fewer vessels increase the hydraulic resistance of the leaf, thereby reducing K_leaf_ (Aasamaa et al., 2005; Jafarikouhini and Sinclair, 2023). Our study identified four key anatomical determinants, namely, upper epidermal cell thickness, xylem area in leaf/petiole tissues, vessel diameter, and vascular bundle frequency, that exhibited significant positive correlations with K_leaf_ (**Figures 8**, **9**; **Supplementary Table S4**). Notably, xylem vascular architecture emerges as the principal modulator of leaf hydraulic vulnerability under mild-to-moderate drought (Bouche et al., 2015; Trifiló et al., 2016). Under severe drought conditions, xylem embolism can lead to irreversible damage, significantly affecting water transport and leaf hydraulic conductivity (Knipfer et al., 2015). The phenomenon of embolism severely impedes water transport, leading to an increase in hydraulic resistance (Trifiló et al., 2016). Our study observed that under drought stress, both the number and diameter of xylem vessels in the leaves and petioles decreased under the MD and SD treatments (**Tables 1**, **2**), indicating the occurrence of changes in the xylem vessels that resulted in a significant reduction in K_leaf_ (**Figure 3A**). Although embolism quantification remains technically challenging in herbaceous species, the observed structural degradation under SD conditions suggests its detrimental role in K_leaf_ reduction. Future studies should prioritize non-destructive embolism detection techniques to elucidate their long-term impacts on hydraulic performance and refine our understanding of drought adaptation strategies in crops.

Aquaporins, which are pivotal regulators of plant water homeostasis, mediate critical physiological processes, including transmembrane water transport and stress responses. Specifically, the decrease in aquaporins gene expression directly affected K_leaf_, as aquaporins play a key role in water transport in chloroplasts and vascular sheath cells (Maurel et al., 2016). It is generally believed that under drought stress conditions, plants maintain their internal water by downregulating aquaporin synthesis gene expression levels and reducing aquaporin content (Afzal et al., 2016). In this study, the aquaporin content under the CK treatment was significantly higher than under the MD and SD treatments (**Figure 4A**). This aligns with the results of Šurbanovski et al. (2013), who demonstrated drought intensity-dependent suppression of *PIP* isoforms, and Xue et al. (2021), who reported coordinated *PIP/TIP* depression in drought-stressed strawberry. In this study, under the MD treatment, the expression of *PIP*-related synthesis genes in cotton leaves were down-regulated by 1.0–1.5-fold, while under the SD treatment, it decreased by 2.0–2.5-fold. Furthermore, under both drought treatments, the expression of *TIP*-related synthesis genes also decreased by 1.0–1.5-fold (**Figure 4B**). These results suggest that cotton adjusts aquaporin gene expression in its leaves to regulate the aquaporin content, thereby controlling the water transport efficiency. Thus, changes in aquaporins genes expression are not only an important molecular marker of cotton’s response to drought stress, but also a key driver of its reduced hydraulic efficiency and photosynthetic performance. Future studies could regulate the expression of aquaporins through gene editing or transgenic techniques to explore their potential in improving drought tolerance in cotton.

### 4.3 Decrease in leaf hydraulic conductivity under drought stress leads to decreases in photosynthetic functions

Green plants are confronted with a contradictory challenge—maximizing absorption of carbon dioxide for photosynthesis while minimizing water loss. Stomatal regulation plays a crucial role in this process (Harayama et al., 2019). Stomata not only regulate the entry of carbon dioxide but also control water evaporation. Therefore, stomatal behavior is vital for plant responses to water stress. Typically, plants regulate g_s_ by changing stomatal size or stomatal density, which allows them to maintain growth and physiological activities under constantly changing environmental conditions (Fang et al., 2019). This experiment demonstrated that g_s_ was significantly and positively correlated with stomatal width, stomatal area, and stomatal aperture, but it showed a highly significant negative correlation with stomatal density (**Supplementary Figure S4A–D**). These results suggest that changes in stomatal traits directly affect stomatal conductance, thereby influencing the plant’s photosynthetic capacity. Under drought conditions, leaves with smaller stomatal sizes and higher densities are more sensitive to changes in the external environment, with faster opening and closing speeds, which effectively reduce water loss and enhance water use efficiency (Raven, 2014; Kardiman and Ræbild, 2017). In this study, MD and SD treatments decreased stomatal width by 6.83% and 13.37%, respectively, and stomatal aperture by 20.59% and 27.58%, respectively, and K_leaf_ significantly decreased under both treatments (**Figure 3A**). These findings suggest that plants reduce water loss and maintain growth by decreasing stomatal size and increasing stomatal density.

Previous studies across various species have shown that under short-term environmental changes, g_s_ and K_leaf_ exhibit a positive correlation, suggesting that K_leaf_ is a potential trigger for the decrease in g_s_ (Theroux Rancourt et al., 2015; Xiong et al., 2018; Wang et al., 2018). Gleason et al. (2017) found that the plant’s water supply capacity weakened as drought stress intensified, decreasing K_leaf_, which triggered stomatal closure, reduced the photosynthetic rate, and improved the water use efficiency in the leaves. This further confirms that K_leaf_ is significantly positively correlated with stomatal aperture and width (**Figure 9**; **Supplementary Table S4**), indicating that as K_leaf_ decreases, the stomatal width and aperture also decrease, directly affecting the plant’s photosynthetic function. In addition, K_leaf_ had an extremely significant positive correlation with g_s_ and A_n_ under different drought treatments (**Figures 10A, B**). These findings suggested that drought stress affected hydraulic signaling by reducing K_leaf_, which triggers rapid stomatal closure and a decrease in aperture, ultimately reducing water loss. However, this adaptive mechanism also decreases photosynthesis.

While our study provides valuable insights into the physiological and anatomical responses of cotton leaves to drought stress using potted plants in a greenhouse, it is important to acknowledge some limitations. The experimental setup, while allowing precise control of conditions, may not fully replicate field complexities. Consequently, yield and quality traits, which are critical for assessing the long-term impact of drought stress on cotton production, were not measured. Future research should extend these findings to field trials and explore the impacts of prolonged drought.

## 5 Conclusion

Our findings demonstrated that drought stress significantly reduced K_leaf_ in cotton by altering leaf anatomical traits, such as decreasing xylem vessel diameter, number, and area, as well as aquaporin content. The decline in K_leaf_ was closely associated with reductions in stomatal aperture, g_s_, and A_n_, which impaired plant growth. Adaptive responses, such as increased VLA_major_ and reduced leaf area, mitigated water loss under drought conditions. Notably, the strong correlation between K_leaf_ and VLA_minor_ highlighted its critical role in maintaining hydraulic efficiency and photosynthetic function. Strategies aimed at improving K_leaf_, such as optimizing xylem morphology and increasing VLA_minor_, could enhance drought tolerance in cotton.

## Data Availability

The original contributions presented in the study are included in the article/**Supplementary Material**, further inquiries can be directed to the corresponding author/s.
