# Supplementary material for: Leaf hydraulic decline coordinates stomatal and photosynthetic limitations through anatomical adjustments under drought stress in cotton
Source: Front Plant Sci. 2025 Jul 10;16:1622308. doi: 10.3389/fpls.2025.1622308 (PMC12287040; doi:10.3389/fpls.2025.1622308)
Supplement: Supplementary file 1 [file DataSheet1.docx]

**Supplementary Figures**


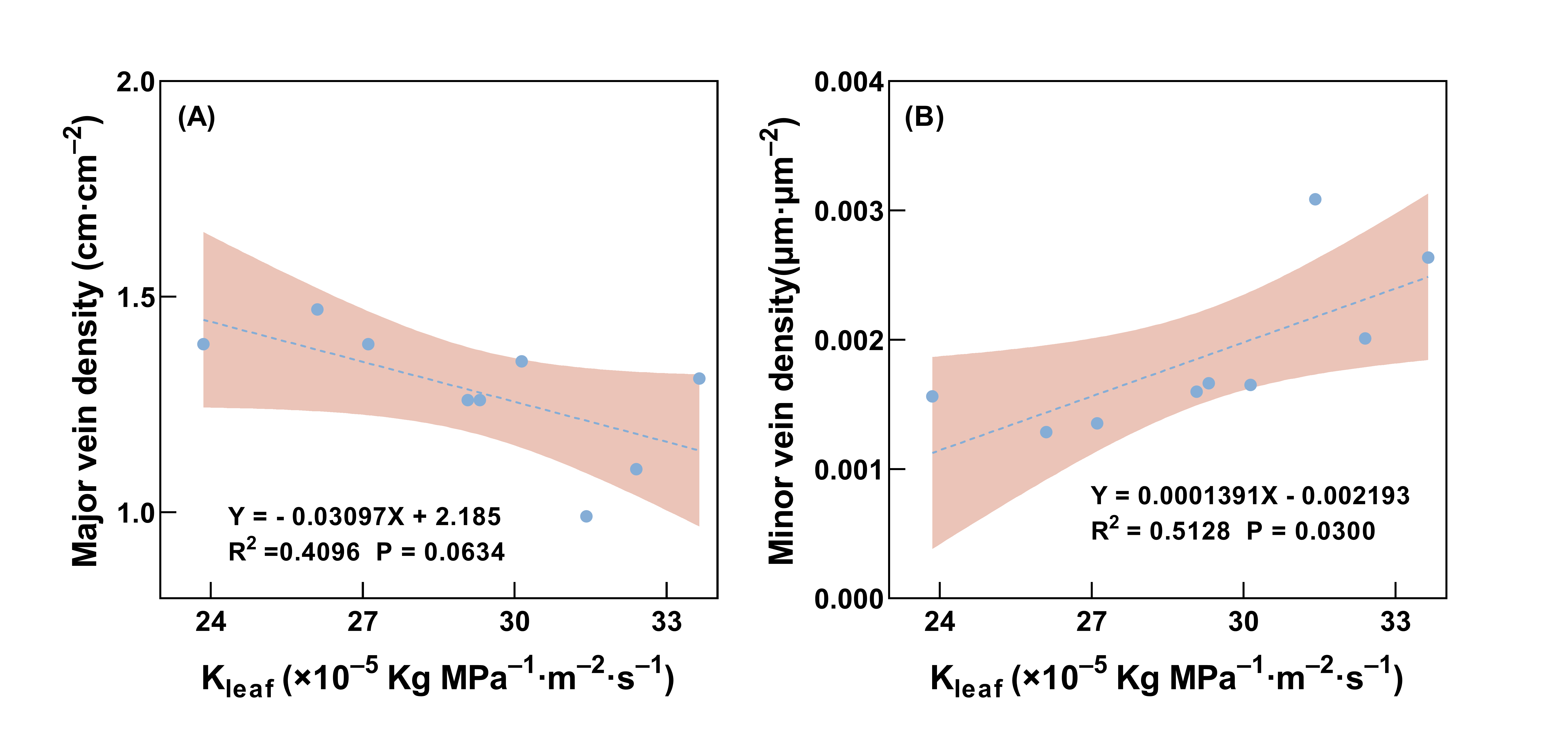


**Supplementary Figure 1.** Linear regression models between K_leaf_ and the density of primary veins (A) as well as the density of secondary veins (B).


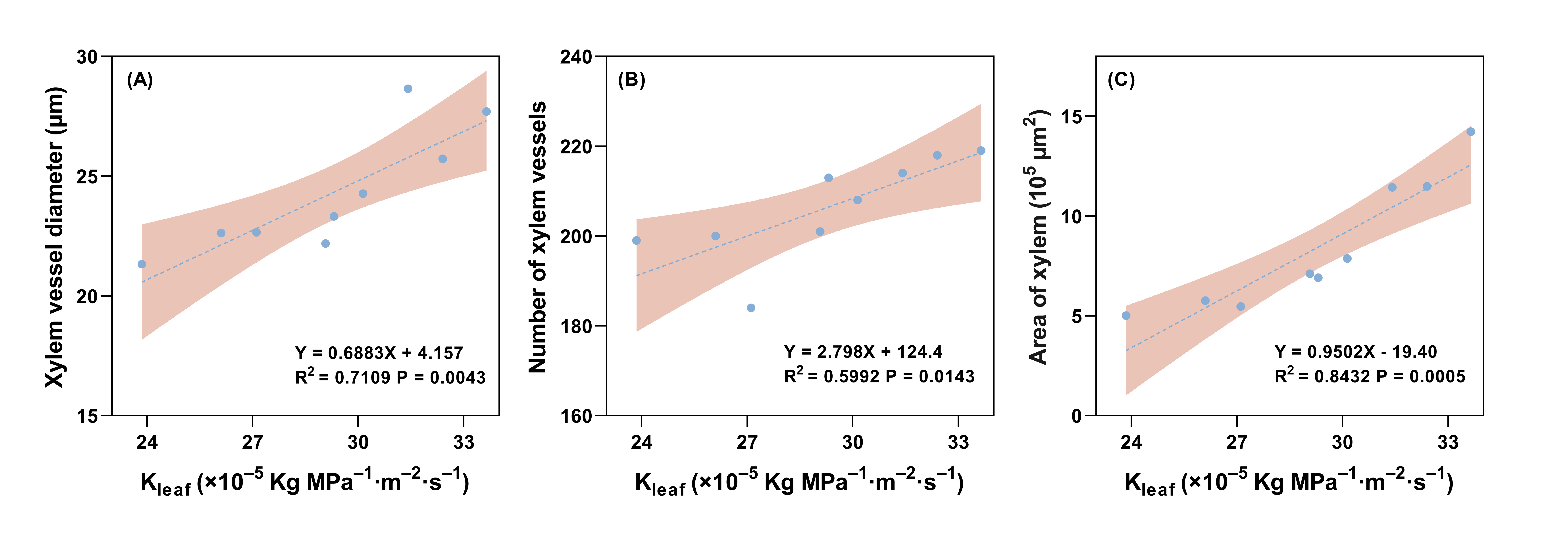


**Supplementary Figure 2.** Linear regression model between K_leaf_ petiole xylem catheter diameter (A), xylem catheter number (B), and area of xylem (C).


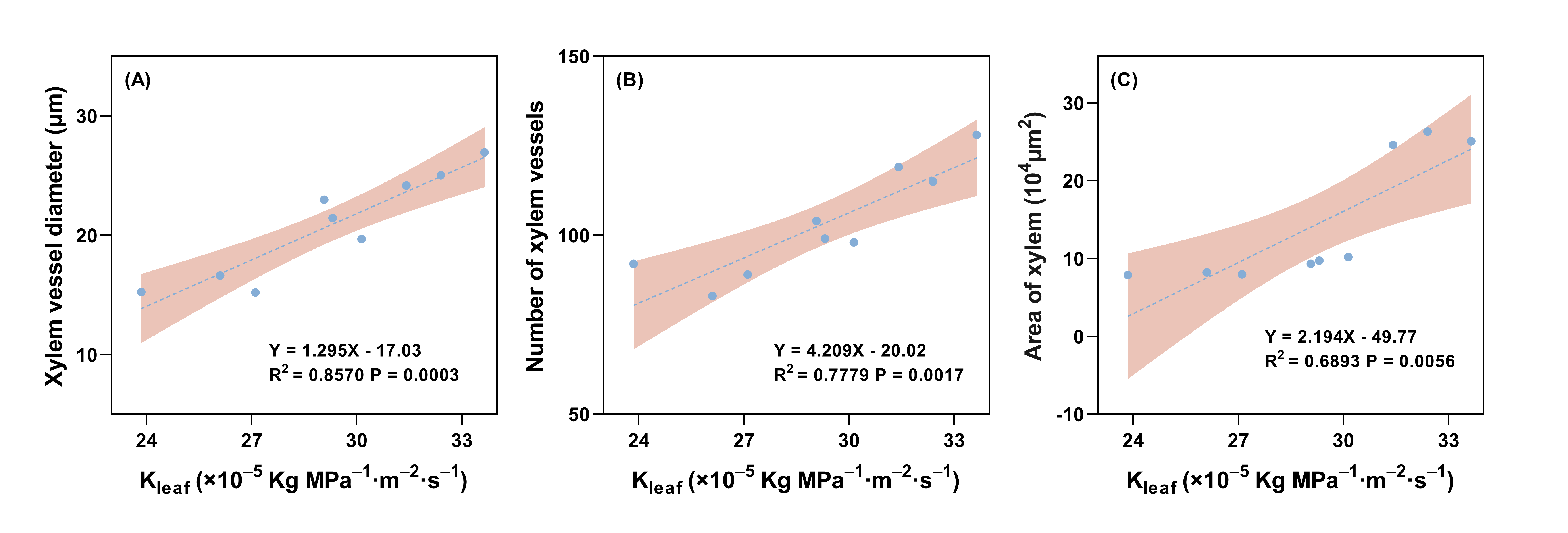
**Supplementary Figure 3.** Linear regression models of K_leaf_ versus the diameter of xylem vessels in leaves (A), the number of xylem vessels (B) and the area of xylem (C).


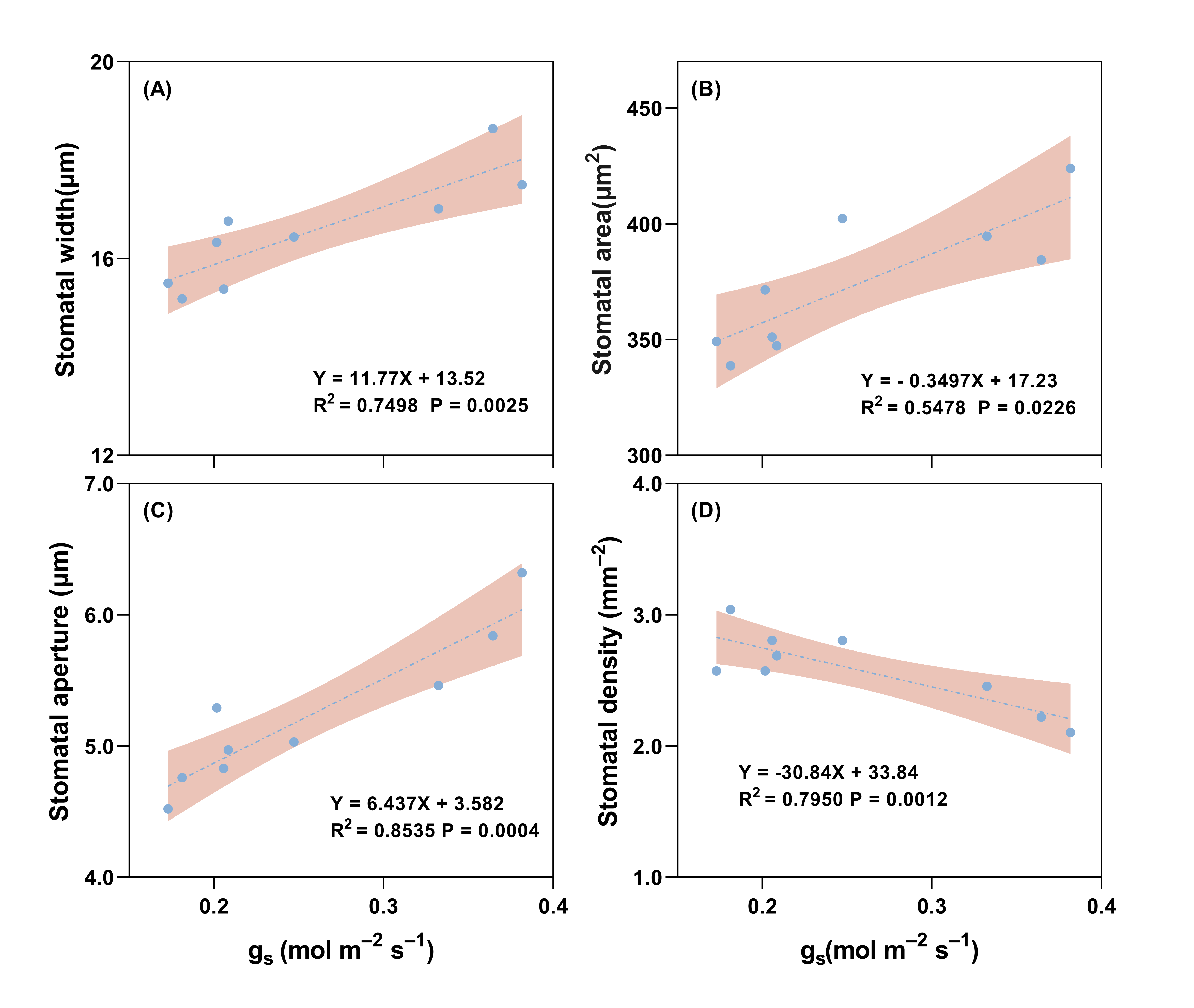


**Supplementary Figure 4.** Linear regression models of stomatal conductance versus stomatal width (A), stomatal area (B), stomatal density (C) and stomatal pore size (D).

**Supplementary Tables**

Total RNA of cotton was extracted with EASY spin Plus Plant RNA Kit (Alid lab company USA). Prime Script reverse transcription kit is reverse transcribed into cDNA, and the inversion system is as follows:

**Table S1**

Reverse transcription reaction system

| Ingredient | content |
| --- | --- |
| RNase free dd H_2_O | 16 µL |
| 4 × gDNA Wiper Mix | 4 µL |
| RNA | 2 µg |
| 42 ℃ | 2 min |
| 5 × qRT Super Mix | 4 µL |
| 50 ℃ | 15 min |
| 85 ℃ | 5 sec |

Primer-primer 6.0 software was used to design primers (Primer Premier, Canada), and the sequence primers are shown in Table S3, in Quant. Studio 5 carries out RT-QCPR reaction, and the reaction system is as follows:

**Table S2**

RT-qPCR reaction system

| Ingredient | content |
| --- | --- |
| AceQ qPCR SYBR Green Master Mix | 10 µL |
| Primer | 0.5 µL |
| cDNA | 1 µg |
| Deionized H_2_O | 8.5µL |
| Cycle setting condition | |
| 95 ℃ | 2 min |
| 95 ℃ | 5 sec |
| 60 ℃ | 30 sec |

Finally, the relative expression of the gene was calculated by 2^-△△Ct^ method.

**Table S3**

Primers of aquaporin gene and internal reference gene used in real-time PCR experiment

| Primer name | Primer sequence |
| --- | --- |
| *Gh_TIP1-3* qF | GCCTTTGCCTTGTTTGTGG |
| *Gh_TIP1-3* qR | GGATACACCCGAGGATAGCG |
| *Gh_TIP1-2* qF | ACATCTTGGCTGGTGGTGC |
| *Gh_TIP1-2* qR | TGTAGTGGGAAGCTGTTCGTG |
| *Gh_PIP2-1* qF | AGTTCCAAGCCAAGGACTACCA |
| *Gh_PIP2-1* qR | TGCCAACACCACCGCATT |
| *Gh_PIP2-2* qF | AGCACTGGCACTGGTTTGG |
| *Gh_PIP2-2* qR | GGCTTGTCCTGGTTGAACATAA |

**Table S4**

Pearson correlation analysis of K_leaf_ with the anatomical structures of leaves and petioles, stomatal characteristics and leaf veins.

|  | K_leaf_ |  | K_leaf_ |
| --- | --- | --- | --- |
| VLA_major_ | -0.64003 | Stomatal length | 0.60422 |
| VLA_minor_ | 0.71609* | Stomatal width | 0.91428*** |
| Thickness of upper epidermis | 0.93212*** | Stomatal area | 0.77519* |
| Thickness of lower epidermis | -0.83557** | Stomatal density | -0.85492** |
| Thickness of Palisade tissue | -0.62613 | Stomatal aperture | 0.8718** |
| Thickness of Spongy tissue | 0.88414** | Cross Sectional Area | 0.93552*** |
| Area of phloem | 0.80281** | Area of phloem | 0.71013* |
| Area of Xylem | 0.83027** | Area of Xylem | 0.91826*** |
| Area of Middle | 0.8201** | Epidermal cell thickness | 0.65888 |
| Number of xylem vessels | 0.88198** | Number of xylem vessels | 0.77409* |
| Xylem vessel diameter | 0.92576*** | Xylem vessel diameter | 0.84316** |

Values are means ±SE (n=3).
